# A Year in the Shadow of Terror: Longitudinal Effects of the October 7, 2023 Terrorist Attack on PTSD, Depression, Anxiety, and Suicidal Ideation Across Distinct Exposure Groups

**DOI:** 10.1192/j.eurpsy.2026.10683

**Published:** 2026-07-31

**Authors:** Y. Levi-Belz

**Affiliations:** university of Haifa, Haifa, Israel

## Abstract

**Introduction:**

On October 7, 2023, Israel experienced a large-scale terrorist attack followed by a prolonged war, exposing civilians and military personnel to acute and sustained trauma. While prior studies have documented short-term psychological effects of mass trauma, few have included baseline assessments or addressed long-term trajectories across distinct exposure groups. In this study, we aimed to examine changes over time in both probable diagnoses and symptom severity of PTSD, depression, anxiety, and suicidal ideation (SI), while accounting for pre-attack symptom levels among different exposed group.

**Objectives:**

In this study, we aimed to examine changes over time in both probable diagnoses and symptom severity of PTSD, depression, anxiety, and suicidal ideation (SI), while accounting for pre-attack symptom levels among different exposed groups.

**Methods:**

A prospective, representative study assessed 614 Israeli participants (309 females; 50.3%) through an online survey conducted across three timepoints: prior to the attack (T1), one month after (T2), and one year later (T3). Participants were categorized into four mutually exclusive exposure groups based on a predefined hierarchy prioritizing the most impactful exposure: direct exposure, bereavement (loss of a close other), reserve-duty combatants and indirect exposure. Probable diagnoses of PTSD (using the ITQ), depression (PHQ-2) and anxiety (GAD-2) were assessed alongside with symptom severity and suicidal ideation (SI by the C-SSRS). Generalized Estimating Equations (GEE) were used to examine main and interaction effects of exposure type and time (T2 to T3), controlling for baseline symptom levels (T1).

**Results:**

Overall, prevalence and severity of psychiatric symptoms declined between T2 and T3. However, exposure group moderated these changes. Reserve-duty combatants exhibited the highest rates of probable diagnoses and symptoms at both time points, with minimal improvement over time. In contrast, indirectly exposed participants demonstrated significant symptom reduction. Uniquely, SI increased over time among reserve-duty participants, highlighting their vulnerability

**Conclusions:**

Recovery following mass trauma such as October 7^th^ attack is not uniform. Exposure type and initial distress levels shape distinct psychological trajectories. Findings underscore the importance of differentiated, long-term, and trauma-informed interventions—especially for bereaved and reserve-duty individuals. Integration of baseline mental health data enhances risk identification and has critical implications for both clinical care and policy planning in the context of ongoing national crises.

**Disclosure of Interest:**

None Declared

